# Prevalence of carbapenemase production among *Enterobacterales* with reduced susceptibility to meropenem in patients at Hospital de Clínicas de Porto Alegre (HCPA) between 2019 and 2024

**DOI:** 10.1007/s42770-026-01995-9

**Published:** 2026-07-01

**Authors:** Eduarda Lanzoni Filter, Mariana Preussler Mott, Karine Rigon Zimmer, Denise Pires Machado, Larissa Lutz, Caroline Constante, Rodrigo Minuto Paiva

**Affiliations:** 1 Unidade de Microbiologia, Serviço de Diagnóstico Laboratorial, Hospital de Clínicas de Porto Alegre (HCPA), Porto Alegre, Brazil; 2 Universidade Federal de Ciências da Saúde de Porto Alegre (UFCSPA), Porto Alegre, Brazil

**Keywords:** Antimicrobial resistance, *Enterobacterales*, Carbapenemases, Meropenem, Hospital environment

## Abstract

Antimicrobial resistance (AMR) represents one of the major threats to global public health, particularly in hospital settings. Among the most concerning pathogens are carbapenemase-producing *Enterobacterales* (CPE), which compromise the effectiveness of last-line antibiotics such as meropenem. This study aimed to analyze the prevalence of carbapenemase production among *Enterobacterales* classified as resistant or susceptible, increased exposure to meropenem at the Hospital de Clínicas de Porto Alegre (HCPA) between 2019 and 2024. This descriptive, retrospective study was based on laboratory data obtained through a structured query of the microbiology database, without access to clinical or identifiable patient information. Carbapenemase detection was performed using the NG-Test^®^ Carba 5 and high-resolution melting quantitative polymerase chain reaction (HRM-qPCR). Data were anonymized and analyzed using absolute and relative frequencies. Temporal analysis revealed a shift in the carbapenememase profile over the study period, characterized by a progressive decline in KPC-producing isolates in the post-pandemic years and a concomitant increase in NDM-producing *Enterobacterales*, indicating a significant epidemiological change. This study contributes to understanding the evolving epidemiology of CPE and supports improved surveillance and clinical management strategies in the context of AMR.

## Introduction

Antimicrobial resistance (AMR) is defined by the World Health Organization (WHO) as the ability of microorganisms to adapt when exposed to antimicrobial agents, ultimately rendering these drugs ineffective [1]. AMR is currently one of the most pressing global public health challenges [2]. In hospital settings, AMR poses a particularly serious threat, as infections caused by resistant organisms complicate treatment and increase morbidity and mortality. In 2019, bacterial AMR was associated with an estimated 4.95 million deaths worldwide, including 1.27 million directly attributable to infections by resistant microrganisms [3].

Among the pathogens of greatest concern are carbapenemase-producing *Enterobacterales* (CPE), which undermine the efficacy of carbapenems, such as meropenem, commonly used as last-line therapeutic agents [4]. Therapeutic options for these infections are limited because carbapenemases hydrolyze most β-lactam antibiotics. Newer agents such as ceftazidime–avibactam (CAZ-AVI) have emerged as promising alternatives; however, resistance to these drugs may also develop, reinforcing the need for continuous surveillance and detailed epidemiological monitoring [4, 5]. CAZ-AVI is active against class A carbapenemases (e.g., *Klebsiella pneumoniae* Carbapenemase (KPC)) and some class D enzymes (e.g., Oxacillinase-48 (OXA-48)), but lacks activity against metallo-β-lactamases (MBLs) such as New Delhi Metallo-beta-lactamase (NDM), Verona Integron-encoded Metallo-β-lactamase (VIM) and Imipenemase metallo-β-lactamase (IMP) [5].

During the COVID-19 pandemic, Brazil reported a marked increase in antimicrobial-resistant *Enterobacterales*, with carbapenem resistance rising from 42% to 58% between 2019 and 2021 [6]. In response to this global scenario, the WHO classified CPE as a “critical priority” pathogen group in 2024, emphasizing the urgent need for research and improved strategies to prevent and control AMR [1]. Approximately 85% of Carbapenem-resistant *Enterobacterales* worldwide produce carbapenemases, significantly limiting therapeutic options and contributing to prolonged treatment durations, higher healthcare costs and increased toxicity when compared with infections caused by carbapenem-susceptible strains [7].

In this context, for a reference hospital such as the Hospital de Clínicas de Porto Alegre (HCPA), assessing the prevalence of carbapenemase-producing *Enterobacterales* with reduced susceptibility to meropenem is essential, given the changes observed in the global and national epidemiological scenario. Recent years have been marked by an increase in metallo-β-lactamases, as well as in the co-production of different carbapenemases, which represents a major therapeutic challenge, since agents such as CAZ-AVI are ineffective against metallo-β-lactamase–producing isolates [8–11]. Therefore, monitoring the local prevalence of these resistance mechanisms is crucial to guide antimicrobial stewardship strategies, infection control measures, and clinical decision-making. The objective of this study was to evaluate the prevalence of carbapenemase production among *Enterobacterales* with reduced susceptibility to meropenem isolated from patients at HCPA between 2019 and 2024.

## Materials and methods

This was a descriptive, retrospective study based on laboratory data from patients treated at the HCPA, a tertiary teaching hospital in southern Brazil, between January 1st, 2019 and December 31st, 2024.

A total of 4232 *Enterobacterales* isolates with reduced susceptibility to meropenem were included. Reduced susceptibility was defined as isolates categorized as *Resistant* or *Susceptible*,* increased exposure* according to the Brazilian Committee on Antimicrobial Susceptibility Testing (BRCAST) criteria in effect at the time of isolation.

All data were obtained through an anonymized query of the microbiology laboratory database (Microbiology Unit, Laboratory Diagnostic Service - SDLab), including bacterial identification, antimicrobial susceptibility profiles, and carbapenemase detection results.

Bacterial identification was performed using MALDI-TOF mass spectrometry (Vitek^®^MS, Biomerieux, France) Antimicrobial susceptibility testing was conducted by disk diffusion using 10 µg meropenem disks in accordance with the current BRCAST.

Carbapenemase detection was performed using either the NG-Test Carba 5^®^, an immunochromatographic assay capable of detecting the five main carbapenemases (KPC, NDM, VIM, IMP and OXA-48-like), or by high-resolution melting quantitative PCR (HRM-qPCR), assays that targeted the main carbapenemase genes, including *bla*_KPC_, *bla*_NDM_, *bla*_VIM_, *bla*_IMP_, *bla*_GES_ and *bla*_OXA-48-like_.

The selection of the detection method was based on the routine workflow of the microbiology laboratory during each period of the study. Therefore, not all isolates were tested using both methodologies.

Results classified as “negative” correspond to isolates in which no carbapenemase was detected by the method employed, and do not exclude the presence of carbapenemases not covered by the assay.

Clinical and microbiological data were organized in a spreadsheet (Google Sheets) and analyzed using descriptive statistics. Absolute and relative frequencies of all relevant variables were calculated. Data analysis was conducted using SPSS (Statistical Package for the Social Sciences). To compare proportions of carbapenemase types (e.g., KPC and NDM) across study years and across species, chi-square (χ²) tests were applied, considering a significance level of *p* < 0.05.

This study was approved by the Research Ethics Committee of the HCPA (CAAE: 92768425.7.0000.5327). All data were obtained through an anonymized database query, with no access to identifiable patient information, the requirement for informed consent was waived. All procedures were conducted in accordance with the ethical standards of the institutional research committee.

## Results

Between 2019 and 2024, a total of 4232 tests for the detection and identification of carbapenemases were performed using the NG-Test Carba 5^®^ and HRM-qPCR methodologies in *Enterobacterales* isolates resistant or susceptible, increased exposure to meropenem at the HCPA. The annual number of tests varied over the study period, with 452 tests in 2019, 582 in 2020, 1,034 in 2021, 770 in 2022, 667 in 2023, and 727 in 2024.

As shown in Table 1, *Klebsiella pneumoniae* was the most frequently identified species among the analyzed isolates, accounting for 3058 samples (72.3%) during the study period. The next most common species were *Serratia marcescens* and *Enterobacter* spp., with 295 (7.0%) and 216 (5.1%) isolates, respectively. Although *K. pneumoniae* and *S. marcescens* represented the majority of KPC-producing isolates, other species - including *Enterobacter* spp., *Escherichia coli*, *Morganella morganii*, and *Proteus mirabilis* - also demonstrated carbapenemase production, particularly of the NDM type.

**Table 1:** Frequency of *Enterobacterales* species and carbapenemase types identified in bacterial isolates from HCPA between 2019 and 2024

BACTERIA	CARBAPENEMASE	QUANTITY	TOTAL
*Citrobacter amalonaticus*	KPC	1	1
*Citrobacter braakii*	NDM	7	8
	KPC	1	
*Citrobacter freundii*	NDM	40	52
	KPC	4	
	OXA-48	2	
	KPC + NDM	2	
	NEGATIVE	2	
	NDM + OXA-48	2	
*Citrobacter koseri*	NDM	2	2
*Citrobacter species*	NDM	13	14
	KPC	1	
*Citrobacter werkmanii*	KPC	1	1
*Citrobacter youngae*	NDM	1	1
*Enterobacter aerogenes*	NDM	1	1
*Enterobacter cloacae*	NDM	3	5
	OXA-48	1	
	NDM + OXA-48	1	
*Enterobacter hormaechei*	NDM	59	102
	OXA-48	13	
	KPC	10	
	KPC + NDM	9	
	NDM + OXA-48	7	
	NEGATIVE	4	
*Enterobacter kobei*	NDM	2	3
	NEGATIVE	1	
*Enterobacter sp.*	KPC	39	216
	OXA-48	29	
	NDM	96	
	NEGATIVE	23	
	KPC + NDM	15	
	NDM + OXA-48	13	
	GES + NDM	1	
*Escherichia coli*	NDM	148	199
	KPC	33	
	NEGATIVE	11	
	KPC + NDM	5	
	IMP	1	
	NDM + VIM	1	
*Escherichia hermanii*	NDM	1	1
*Klebsiella aerogenes*	KPC	10	22
	NEGATIVE	6	
	NDM	6	
*Klebsiella oxytoca*	NDM	54	71
	KPC	10	
	NEGATIVE	5	
	KPC + NDM	2	
*Klebsiella pneumoniae*	KPC	1853	3058
	NDM	658	
	KPC + NDM	415	
	NEGATIVE	86	
	OXA-48	25	
	NDM + OXA-48	11	
	KPC + OXA-48	9	
	IMP	1	
*Klebsiella sp.*	NDM	2	6
	KPC	2	
	NEGATIVE	2	
*Klebsiella variicola*	NDM	4	5
	OXA-48	1	
*Kluyvera ascorbata*	KPC	1	1
*Leclercia adecarboxilata*	NDM	1	1
*Morganella morganii*	NDM	61	66
	KPC	3	
	NEGATIVE	2	
*Pluralibacter gergoviae*	KPC	2	3
	NDM	1	
*Proteus mirabilis*	NDM	46	50
	NEGATIVE	2	
	NDM + VIM	1	
	KPC	1	
*Proteus sp.*	NDM	2	2
*Proteus vulgaris*	NDM	3	3
*Providencia rettgeri*	NDM	16	18
	NDM + IMP + GES	1	
	KPC	1	
*Providencia species*	NDM	1	1
*Providencia stuartii*	NDM	19	21
	NDM + OXA-48	1	
	NEGATIVE	1	
*Raoultella ornithinolytica*	NDM	2	2
*Serratia marcescens*	KPC	255	295
	NDM	18	
	NEGATIVE	12	
	KPC + NDM	10	
*Serratia sp.*	KPC	1	1

“Negative” indicates that no carbapenemase was detected by the method used (NG-Test Carba 5^®^ or HRM-qPCR), and does not exclude the presence of carbapenemases not targeted by the assay

*KPC* Klebsiella pneumoniae carbapenemase, *NDM* New Delhi metallo-β-lactamase, *VIM* Verona integron-encoded metallo-β-lactamase, *IMP* Imipenemase metallo-β-lactamase*,* *OXA-48* Oxacillinase-48

Table 2 presents the overall distribution of carbapenemases identified in *Enterobacterales* isolates with resistant or susceptible, increased exposure to meropenem at HCPA. During the study period, KPC was the predominant carbapenemase, detected in 52.7% of isolates, followed by NDM (29.9%) and KPC + NDM co-producers (10.8%). Enzymes such as OXA-48 and other multiple combinations (NDM + OXA-48, IMP, VIM, GES) occurred sporadically, accounting for less than 2% of cases.

Temporal analysis demonstrated a progressive decrease in KPC, which declined from 76.3% in 2019 to 43.0% in 2024, corresponding to the post-pandemic period. In contrast, NDM showed a marked increase, rising from 16.4% in 2019 to 35.2% in 2024, indicating a relevant epidemiological shift. Additionally, an increase in the proportion of KPC and NDM co-producing isolates was observed, reaching 14.7% in 2024, suggesting potential genetic recombination events or dissemination of multidrug-resistant plasmids.

**Table 2:** Frequency of carbapenemases identified between 2019 and 2024 at HCPA

YEAR		KPC	NDM	KPC + NDM	NEGA- TIVE	OXA-48	NDM + OXA-48	KPC + OXA-48	IMP	NDM + VIM	NDM + IMP + GES	GES + NDM
2019	n	345	74	14	16	2	0	0	1	0	0	0
	%	76.3	16.4	3.1	3.5	0.4	0	0	0.2	0	0	0
2020	n	408	117	18	29	9	1	0	0	0	0	0
	%	70.1	20.1	3.1	5.0	1.5	0.2	0	0	0	0	0
2021	n	516	275	175	23	37	6	0	0	1	1	0
	%	49.9	26.6	16.9	2.2	3.6	0.6	0	0	0.1	0.1	0
2022	n	357	262	91	30	13	9	8	0	0	0	0
	%	46.4	34.0	11.8	3.9	1.7	1.2	1.0	0	0	0	0
2023	n	290	283	53	21	8	9	1	1	1	0	0
	%	43.5	42.4	7.9	3.1	1.2	1.3	0.1	0.1	0.1	0	0
2024	n	313	256	107	38	2	10	0	0	0	0	1
	%	43.0	35.2	14.7	5.2	0.3	1.4	0	0	0	0	0.1
Total	n	2229	1267	458	157	71	35	9	2	2	1	1
	%	52.7	29.9	10.8	3.7	1.7	0.8	0.2	0.0	0.0	0.0	0.0

The chi-square test results demonstrated a statistically significant association between carbapenemase type and year of detection (*p* < 0.05), confirming the presence of a non-random temporal variation in the distribution of resistance mechanisms. This finding highlights a progressive epidemiological shift at HCPA, characterized by a decrease in KPC and a consistent increase in NDM between 2019 and 2024.

**Fig. 1:**
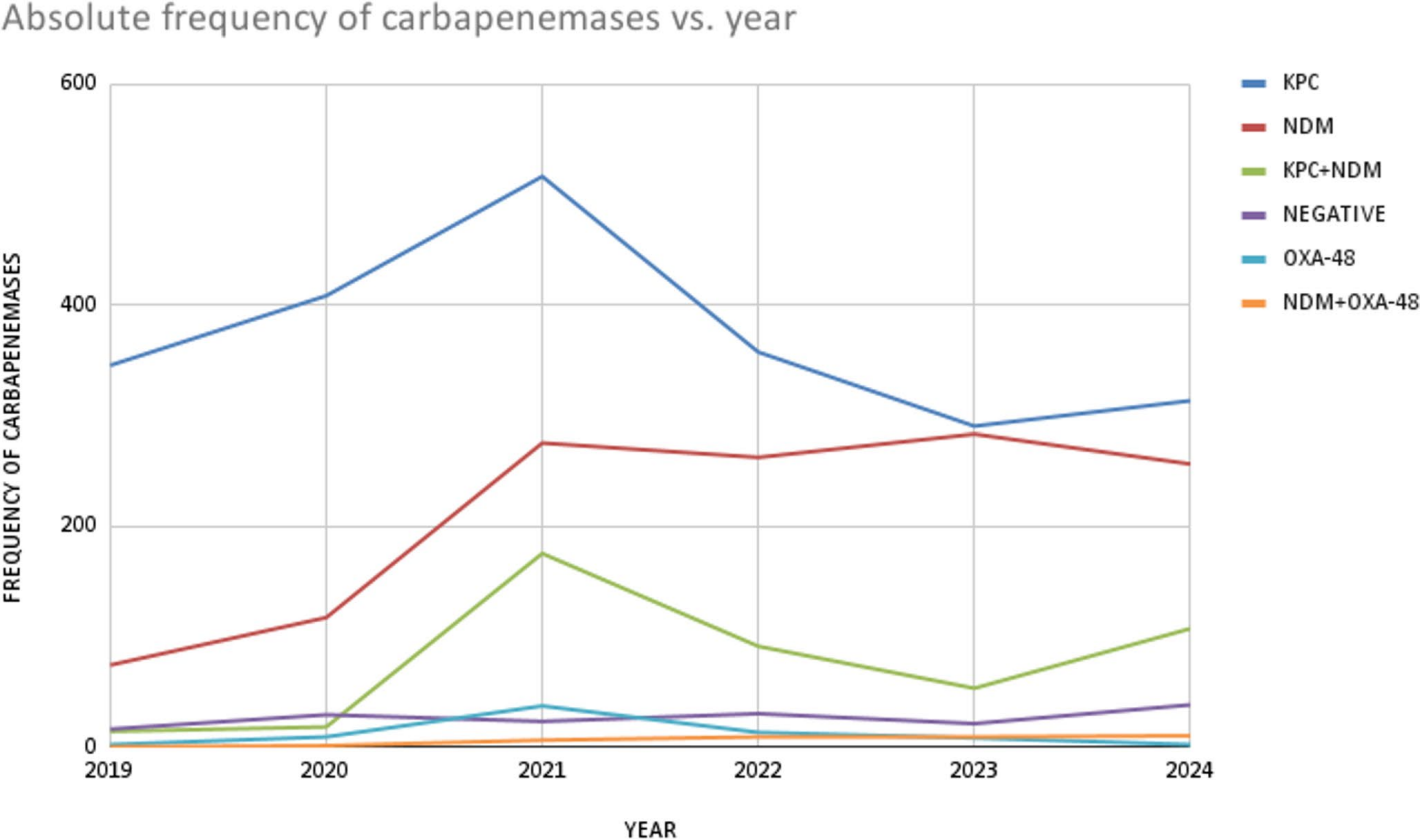
Prevalence of carbapenemases among *Enterobacterales* from 2019 to 2024

Figure 1 shows the annual evolution of the absolute number of isolates positive for each carbapenemase type. A marked increase was observed in 2021, corresponding to the peak detection of KPC (516 isolates) and the rise of NDM (275 isolates). In the subsequent years, there was a reduction in the total number of cases, accompanied by stabilization of the absolute frequencies of KPC and NDM from 2022 to 2024.

## Discussion

According to the WHO’s *Bacterial Priority Pathogens List 2024*, CPE remain among the leading global threats to public health [1]. This resistance is strongly associated with the production of carbapenemase enzymes capable of hydrolyzing and inactivating carbapenem antibiotics [12]. Among the main enzymes, KPC and NDM, known for its high capacity for horizontal dissemination across different bacterial species - are particularly noteworthy [13]. The WHO also highlights that the widespread circulation of these enzymes, especially in low- and middle-income countries, is linked to higher morbidity and mortality rates, therapeutic failures, and increased healthcare costs [1]. In Latin America, Brazil accounts for the highest proportion of carbapenemase-producing isolates, reinforcing the regional magnitude of the problem [14].

This study provides one of the most comprehensive longitudinal analyses of carbapenemase-producing *Enterobacterales* in southern Brazil, covering a six-year period that includes both pre and post-COVID-19 scenarios. Unlike previous reports, this study highlights a clear epidemiological shift within a single tertiary-care center, with a progressive replacement of KPC by NDM, as well as an increase in co-producing isolates. These findings contribute to a better understanding of local resistance dynamics and reinforce the need for continuous molecular surveillance strategies.

Although the results of the present study indicate a predominance of KPC, they also reveal a significant shift in the resistance profile of *Enterobacterales* at HCPA over the evaluated period, marked by a decrease in KPC and a substantial increase in NDM. This trend aligns with national and international findings and reflects an epidemiological reconfiguration in the post-COVID-19 period [6–14]. Although KPC remains the most prevalent carbapenemase in Brazil, the proportion of NDM increased markedly from 1.9% in 2019 to 14.6% in 2022 [15]. These findings corroborate the local results and indicate that NDM is becoming the leading emerging resistance mechanism at HCPA.

At the national level, the multicenter ASCENSION study [16], conducted in 14 Brazilian hospitals, identified *Klebsiella pneumoniae* as the main carbapenemase-producing species (73.8% of resistant isolates), associated with a mortality rate of 42.3%. The study also documented a growing presence of isolates co-producing KPC and NDM (7.2%), a phenomenon attributed to selective pressure resulting from intensive antimicrobial use and prolonged hospital stays during and after the pandemic. Although KPC remains the most prevalent enzyme (64.4%), NDM has gained prominence (28.4%), confirming that the transition observed at HCPA reflects trends described in broader national studies.

The findings of this study are also consistent with previous reports from Latin America and the Caribbean, which analyzed 58,909 carbapenem-resistant bacterial isolates from 12 countries [17]. In that survey, 81.1% of isolates carried carbapenemase genes, with KPC predominating (65.3%), followed by NDM (22.2%) and KPC + NDM co-production in 4.1% of cases. The study also highlighted a significant increase in NDM in the post-pandemic period and substantial geographic heterogeneity, with higher prevalence of NDM in Central America and KPC in South America, illustrating the complexity of the regional resistance landscape.

The horizontal dissemination of NDM across different *Enterobacterales* genera observed in this study has also been reported previously, highlighting the role of intense plasmid-mediated transfer in facilitating the regional spread of this gene [15]. This mechanism explains the increasing detection of NDM in diverse species such as *E. coli*, *S. marcescens*, and *M. morganii*, highlighting the role of mobile genetic elements in diversifying resistance mechanisms. These findings reinforce the importance of ongoing molecular surveillance as an essential tool to monitor and contain the spread of these enzymes.

Carbapenem resistance among *Enterobacterales* represents one of the greatest therapeutic challenges today, as carbapenemase production severely limits treatment options [18]. Consequently, new β-lactam/β-lactamase inhibitor combinations - CAZ-AVI, were approved by the FDA in 2015 and by ANVISA in 2018. A meta-analysis demonstrated improved clinical response, higher microbiological eradication, and reduced mortality in infections caused by KPC-producing *K. pneumoniae* treated with CAZ-AVI compared with other therapeutic regimens [19]. However, the increasing dissemination of NDM, detected across several Brazilian states, reduces the applicability of this drug. The Brazilian Ministry of Health, through Technical Note No. 74/2022-CGLAB/DAEVS/SVS/MS, reported an increase of more than 380% in KPC + NDM co-producing strains between 2018 and 2021, heightening the risk of therapeutic failure [8]. Additionally, 65 CAZ-AVI-resistant KPC variants have been described, underscoring the high adaptive potential of this enzyme and the need for rational antimicrobial use and continuous molecular surveillance [9].

Currently, the only available therapeutic option in Brazil for infections caused by NDM-producing *Enterobacterales* is the combination of CAZ-AVI with aztreonam, as recommended by IDSA guidelines [10] and, more recently, by the Brazilian Society of Infectious Diseases [11], which report significantly reduced mortality compared with alternative regimens. NDM is a metallo-β-lactamase capable of hydrolyzing penicillins, cephalosporins, and carbapenems, rendering these infections extremely difficult to treat [10]. The guidelines also clarify that although aztreonam is not hydrolyzed by NDM, it is often degraded by other β-lactamases co-produced by these bacteria, making avibactam essential to inhibit these additional enzymes and enable treatment efficacy.

Although *Klebsiella pneumoniae* is the main pathogen associated with CPE infections, the results of this study demonstrate that other genera also play a significant role. Among the 3,058 *K. pneumoniae* isolates evaluated, KPC predominated (60.6%), followed by NDM (21.5%) and KPC + NDM co-production (13.6%). *S. marcescens* also showed a predominance of KPC (86.4%), in accordance with the 2024 Epidemiological Bulletin from the Brazilian Ministry of Health, which emphasizes the predominance of KPC in this species [20].

In contrast, among the 216 *Enterobacter* spp. isolates, NDM was the primary resistance mechanism (44.4%), indicating a distinct profile compared with *K. pneumoniae*. Among *E. coli* isolates, 74.4% were NDM producers. In *C. freundii*, NDM was present in 76.9% of isolates. This pattern was also observed among *Morganellaceae*, in which NDM was widely predominant: *M. morganii* (92.4%), *P. mirabilis* (92.0%), *P. rettgeri* (88.9%), and *P. stuartii* (90.5%), indicating a strong association between this group and the dissemination of metallo-β-lactamases.

The treatment of infections caused by *Morganellaceae* (*Morganella*,* Proteus*, and *Providencia spp.*) is particularly challenging due to their intrinsic resistance to multiple antimicrobial agents and the high frequency of metallo-β-lactamase production, especially NDM. In this scenario, the recommended therapeutic option is the combination of CAZ-AVI with aztreonam [10, 11]. However, currently available methods for susceptibility testing of the aztreonam plus ceftazidime–avibactam combination have not been standardized across laboratories, limiting their application in routine clinical practice [21].

NDM was identified in multiple *Enterobacterales* species - including *K. pneumoniae*, *E. cloacae*, *E. coli*, and *C. freundii* - demonstrating its broad capacity for dissemination across genera [15]. This spread is facilitated by the presence of the *bla*_NDM_ gene in mobile genetic elements, particularly plasmids of the IncL/M, IncA/C, IncF, IncHI1, IncN, and IncX3 types, which enable horizontal transfer. Some, such as IncA/C plasmids, have a broad host range and can replicate even in bacteria outside the *Enterobacterales* family, further enhancing dissemination [15]. This combination of high genetic mobility and broad enzymatic activity explains the rapid global spread of NDM, undermining the effectiveness of modern antimicrobials such as CAZ-AVI, which is inactive against metallo-β-lactamases, and significantly reducing available therapeutic options.

In Brazil, NDM was first detected in 2013 in a *P. rettgeri* strain isolated from infected soft tissue in a patient with diabetes in Porto Alegre, Rio Grande do Sul [22, 23]. Following this initial detection, NDM-producing bacteria quickly became recognized as a potential public health threat in the country [23].

In southern Brazil, the *bla*_NDM_ gene has been identified in species belonging to the *Morganellaceae* family, as well as in *K. pneumoniae* and *E. coli* [16]. The occurrence of this gene in these bacteria is associated with broad-host-range plasmids, such as IncA/C, IncHI2, and IncC, which promote interspecies dissemination and reflect the expanding presence of this carbapenemase in the Brazilian context. Among these elements, IncX3 plasmids stand out due to their high conjugation rate, broad compatibility among species, and global distribution across *Enterobacterales*, making them efficient epidemic vectors [24]. In contrast, the *bla*_KPC−2_ gene demonstrates more restricted dissemination because it is typically associated with plasmids of limited compatibility, such as IncF, IncR, and IncN-pST15, linked to epidemic *K. pneumoniae* clones (ST11, ST340, ST307). These plasmids exhibit high stability and persistence but circulate predominantly within the *K. pneumoniae* complex, reducing horizontal transfer to other *Enterobacterales* genera [25].

In summary, the results of this study suggest that HCPA exhibits trends similar to those observed in national and international reports, characterized by the gradual replacement of KPC by NDM and the emergence of co-producing strains. This scenario underscores the need for continuous laboratory surveillance, detailed molecular investigation, and closer integration between clinical microbiology and hospital epidemiology to curb the spread of multidrug-resistant mechanisms and support more effective therapeutic strategies.

## Conclusion

The findings reveal a significant shift in the carbapenem resistance profile among *Enterobacterales* at HCPA, with a reduction in KPC detections and a consistent rise in NDM as the main emerging mechanism. Although KPC remains predominant in Brazil and Latin America, the increasing circulation of NDM - favored by broad-host-range plasmids - indicates a rapidly expanding dissemination process. The identification of co-producing strains further highlights a particularly challenging scenario, especially given the therapeutic limitations imposed by NDM, which restrict treatment primarily to the CAZ-AVI plus aztreonam combination. These results reinforce the importance of ongoing epidemiological surveillance, systematic molecular characterization, and prudent antimicrobial use to limit the spread of multidrug-resistant strains and guide more effective clinical and institutional interventions.

## Data Availability

The datasets generated and analyzed during the current study are available from the corresponding author upon reasonable request.
